# Human Arylamine *N*-Acetyltransferase 1 (NAT1) Knockout in MDA-MB-231 Breast Cancer Cell Lines Leads to Transcription of NAT2

**DOI:** 10.3389/fphar.2021.803254

**Published:** 2022-01-03

**Authors:** Samantha M. Carlisle, Patrick J. Trainor, Mark A. Doll, David W. Hein

**Affiliations:** ^1^ Department of Chemistry and Biochemistry, New Mexico State University, Las Cruces, NM, United States; ^2^ Department of Pharmacology and Toxicology, School of Medicine, University of Louisville, Louisville, KY, United States; ^3^ Division of Cardiovascular Medicine, School of Medicine, University of Louisville, Louisville, KY, United States

**Keywords:** NAT1, arylamine *N*-acetyltransferase 1, RNA-seq, NAT2, arylamine *N*-acetyltransferase 2, cell adhesion, breast cancer

## Abstract

Many cancers, including breast cancer, have shown differential expression of human arylamine *N*-acetyltransferase 1 (NAT1). The exact effect this differential expression has on disease risk and progression remains unclear. While NAT1 is classically defined as a xenobiotic metabolizing enzyme, other functions and roles in endogenous metabolism have recently been described providing additional impetus for investigating the effects of varying levels of NAT1 on global gene expression. Our objective is to further evaluate the role of NAT1 in breast cancer by determining the effect of NAT1 overexpression, knockdown, and knockout on global gene expression in MDA-MB-231 cell lines. RNA-seq was utilized to interrogate differential gene expression (genes correlated with NAT1 activity) across three biological replicates of previously constructed and characterized MDA-MB-231 breast cancer cell lines expressing parental (*Scrambled*), increased (*Up*), decreased (*Down, CRISPR 2–12*), or knockout (*CRISPR 2–19, CRISPR 5–50*) levels of NAT1. 3,889 genes were significantly associated with the NAT1 *N*-acetylation activity of the cell lines (*adjusted p* ≤ 0.05); of those 3,889 genes, 1,756 were positively associated with NAT1 *N*-acetylation activity and 2,133 were negatively associated with NAT1 *N*-acetylation activity. An enrichment of genes involved in cell adhesion was observed. Additionally, human arylamine *N*-acetyltransferase 2 (NAT2) transcripts were observed in the complete NAT1 knockout cell lines (*CRISPR 2–19* and *CRISPR 5–50*). This study provides further evidence that NAT1 functions as more than just a drug metabolizing enzyme given the observation that differences in NAT1 activity have significant impacts on global gene expression. Additionally, our data suggests the knockout of NAT1 results in transcription of its isozyme NAT2.

## 1 Introduction

It is estimated by the American Cancer Society that breast cancer will account for 30% of new cancer cases and 15% of cancer-related deaths in women in 2021 (Siegel et al., 2021). When breast cancer is detected at the localized stage the 5-year survival rate is 99% however when detected at the distant stage the 5-year survival rate is a disappointing 28% (Siegel et al., 2018). These statistics highlight the need for a better understanding of breast cancer risk and progression so that interventions can be provided at the early stage when breast cancer is the most treatable and to identify novel molecular targets for treatment.

Human arylamine *N*-acetyltransferase 1 (NAT1) has been extensively studied in breast cancer. NAT1 is a classically described phase II xenobiotic metabolizing enzyme, but more recently has been shown to have additional, orthogonal, and diverse roles in metabolism including the hydrolysis of acetyl-Coenzyme A (acetyl-CoA) (Laurieri et al., 2014; Stepp et al., 2015; Stepp et al., 2019). There is a strong association between estrogen receptor positive breast cancer and high NAT1 expression (Carlisle and Hein, 2018; Minchin and Butcher, 2018; Zhang et al., 2018). The role of NAT1 in breast cancer, until recently, was thought to center on NAT1’s ability to metabolize/activate carcinogens, however it has been shown that rats with higher Nat2 expression (orthologous to human NAT1) had greater mammary tumor susceptibility, independent of carcinogen metabolism (Stepp et al., 2017).

Additionally, our recent work examining differences in the metabolome between breast cancer cell lines expressing varying levels of NAT1 suggested a role for NAT1 in the metabolism of l-asparagine, putrescine, and l-lysine. We also observed a relationship between NAT1 and the conjugation of carnitine with fatty acyl-CoA moieties (Carlisle et al., 2020). Others have shown deletion of the *NAT1* gene in MDA-MB-231, HT-29 and HeLa cancer cell lines leads to increased collagen adherence, decreased invasion, and morphological changes but no changes to migration ability in all three cell-lines (Li et al., 2020). Despite numerous studies investigating NAT1 in breast cancer, the exact effect of increased NAT1 expression in breast cancer remains unknown. It also remains unknown what additional, diverse roles NAT1 may have in metabolism that are independent of xenobiotic metabolism. In this study our objective was to further investigate the role of NAT1 in pathways commonly dysregulated in cancer.

## 2 Materials and Methods

### 2.1 Description of Cell Line Samples

Six cell lines constructed via both siRNA and CRISPR/Cas9 technologies from the MDA-MB-231 triple negative breast cancer cell line to have varying levels of human NAT1 *N*-acetylation activity were utilized in this study: *Scrambled* (transfection control with parental MDA-MB-231 activity; 9.41 ± 1.15 nmoles acetylated *p*-aminobenzoic acid/min/mg protein), *Up* (stable integration of plasmid overexpressing NAT1 with increased activity; 63.5 ± 10.3 nmoles acetylated *p*-aminobenzoic acid/min/mg protein), *Down* (stable transfection with siRNA specific to NAT1 with decreased activity; 6.55 ± 0.826 nmoles acetylated *p*-aminobenzoic acid/min/mg protein), *CRISPR 2–12* (single allele deletion with CRISPR/Cas9 guide RNA 2 with decreased activity; 5.33 ± 0.572 nmoles acetylated *p*-aminobenzoic acid/min/mg protein), *CRISPR 2–19* (double allele deletion with CRISPR/Cas9 guide RNA 2 with complete knockout of NAT1; no detectable activity), and *CRISPR 5–50* (double allele deletion with CRISPR/Cas9 guide RNA 5 with complete knockout of NAT1; no detectable activity). Details on the construction and characterization of these cell lines has been described in detail elsewhere (Carlisle et al., 2018; Stepp et al., 2019). The *Scrambled*, *Up*, and *Down* cell lines are non-clonal cell lines while the *CRISPR 2–12*, *CRISPR 2–19*, and *CRISPR 5–50* cell lines were established from a single isolated clone following transfection. Additionally, we acknowledge that the *Scrambled* cell line may have changes in gene expression not present in the other cell lines if the randomly generated scrambled shRNA utilized was complementary to anywhere in the genome. This risk was minimized by the chosen design/sequence of the shRNA.

### 2.2 Collection of Samples

**FIGURE 1:**
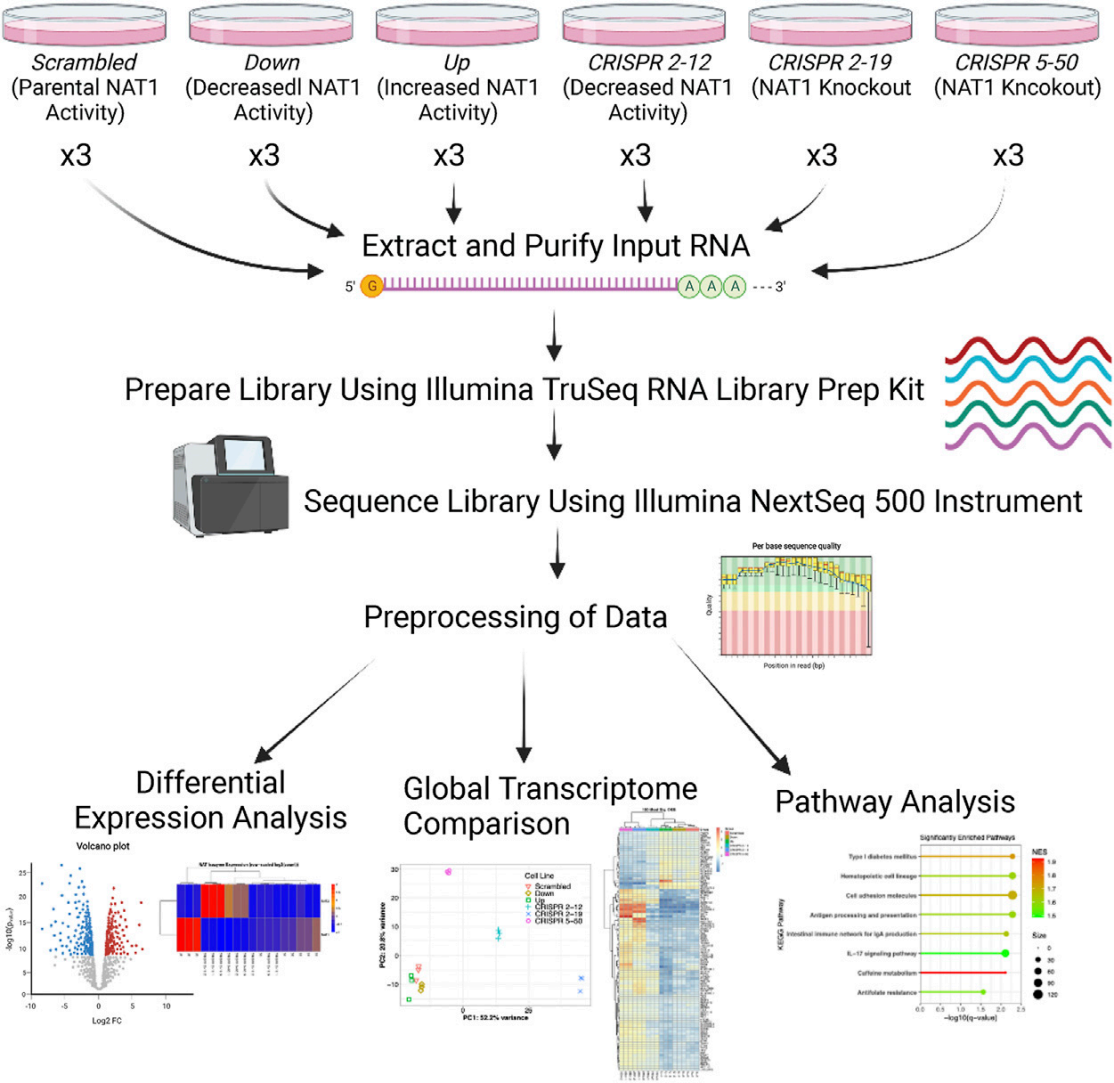
Transcriptomics Experimental Approach. Three biological replicates were collected for each cell line, RNA was extracted and purified, and libraries were prepared using the input RNA. Once library preparation was complete, libraries were sequenced using the Illumina NextSeq 500 instrument. Resulting sequencing data were aligned to the hg38 human reference genome via STAR and read counts were generated for each gene using the Bioconductor R package GenomicAlignments. Next, differential expression analysis, PCA, hierarchical clustering, and pathway analysis was performed.

Figure 1 illustrates the experimental approach and workflow of sample collection described below. Cells were plated in triplicate per biological replicate at a density of 500,000 cells per 150 mm × 25 mm cell plate for both transcriptomics and metabolomics analysis. All cell lines were cultured in high-glucose Dulbecco’s Modified Eagle Medium **(**DMEM), with 10% fetal bovine serum, 5% glutamine, and 5% penicillin/streptomycin added. Cells were allowed to grow for 3 days at 37°C and 5% CO_2_ in an incubator.

Cells were then harvested on ice by adding 5 ml 0.25% trypsin and scraping the cells from the plate. Three plates were combined to form one sample (biological replicate). Three biological replicates for each cell line were collected for transcriptomics analysis. After harvesting the cells, cells were washed 3 times with ice-cold 1 x PBS. All supernatant was removed and 100 μl of cell pellet was reserved for transcriptomics analysis. The remaining cell pellet was reserved for metabolomics analysis. Samples were then flash frozen by placing in a pool of liquid nitrogen for 1 min and stored in an −80°C freezer until RNA isolation.

### 2.3 Transcriptomics

#### 2.3.1 RNA Isolation

Total RNA was isolated from MDA-MB-231 breast cancer cells expressing parental (*Scrambled*), increased (*Up*), decreased (*Down*, *CRISPR 2–12*), and knockout (*CRISPR 2–19, CRISPR 5–50*) levels of NAT1 using the RNeasy^®^ Mini Kit (Qiagen Sciences, Germantown, Maryland) according to manufacturer’s instructions. RNA quality was evaluated and concentrations were measured in each sample using a NanoDrop Bioanalyzer (Thermo Fischer Scientific).

#### 2.3.2 Library Preparation

Libraries were prepared using the TruSeq Stranded mRNA LT Sample Prep Kit- Set A (Illumina, San Diego, California; Cat# RS-122-2101) with poly-A enrichment per manufacturer’s instructions. One µg of total RNA (in a volume of 50 µl) from each sample was used in library preparation. Briefly, the total RNA was fragmented to improve sequence coverage over the transcriptome. Next, the first strand of cDNA was synthesized from the cleaved RNA fragments that were primed with random hexamers using reverse transcriptase and random primers. Then the second strand of cDNA was synthesized, thus giving us double stranded blunt end cDNA. Next, a single ‘A’ nucleotide was added to the 3′ ends of the blunt fragments to prevent them from ligating to one another during the adapter ligation reaction. A corresponding single ‘T’ nucleotide on the 3′ end of the adapter provides a complementary overhang for ligating the adapter to the fragment. Then, multiple indexing adapters were ligated to the ends of the double stranded cDNA, preparing them for hybridization onto a flow cell. Next the DNA fragments were enriched using PCR to selectively enrich those DNA fragments that have adapter molecules on both ends and to amplify the amount of DNA in the library. The PCR was performed with a PCR Primer Cocktail that anneals to the ends of the adapters. Finally, 30 µl of eluted library was collected and stored at −20°C. To avoid skewing the representation of the library, the number of PCR cycles are minimized. This kit includes steps to validate and normalize constructed libraries and methods to check quality control.

#### 2.3.3 Library Validation

Quality: Size, purity and semi quantitation were performed on an Agilent Bioanalyzer using the Agilent DNA 1000 Kit. The final fragment size for all the samples was approximately 300bp which is expected according to the protocol.

Quantity: Sequencing library quantitation was performed by qPCR using the KAPA Library Quantitation Kit for Illumina Platforms. The standard curve method was used for quantitation using one to five DNA standards that came with the kit.

Normalize and Pool Libraries: Ten µl of sample was transferred from the wells to a new MIDI plate. We then normalized the concentration of the libraries to 10 nM using Tris-HCl 10 mM, pH 8.5 with 0.1% Tween 20. Five µl of each sample was then transferred to be pooled into a new LowBind 1.5 ml micro centrifuge tube for a total volume of 60 µl pooled 10 nM library. Then 4 nM dilution was made from the 10 nM pooled library by diluting with Tris-HCl 10 mM, pH 8.5 with 0.1% Tween 20.

Denaturing and diluting Libraries for the Nextseq 500: A total volume of 1.3 ml of 1.8 pM denatured library is needed for sequencing using the v2 kit. Pooled 4 nM library was denatured by mixing with diluted NaOH and incubated at room temperature for 5 min 200 mM Tris HCl, pH 7.0 was then added. The reaction mixture was diluted to 20 pM using a pre-chilled Hybridization Buffer. 20 pM denatured library was further diluted to 1.8 pM using the same Hybridization Buffer. Before loading onto the reagent cartridge, 1.3 µl of denatured 20 pM Phix control was added to the 1,299 µl of denatured 1.8 pM library to a total volume of 1.3 ml for the first sequencing run (for the 2nd sequencing run 1.9 pM library was used).

#### 2.3.4 Sequencing

Sequencing was performed on the University of Louisville Center for Genetics and Molecular Medicine’s (CGeMM) Illumina NextSeq 500 using the NextSeq 500/550 75 cycle High Output Kit v2 (FC-404-2005). A second run was performed to increase the number of reads. For each run, 72 single-end raw sequencing files (.fastq) (Cock et al., 2010) representing six conditions with three biological replicates and four lanes per replicate were generated. Reads were 75 base pair, single-end reads.

#### 2.3.5 RNA-Seq Analysis

Adapter sequences were trimmed from resulting reads using Trim Galore (version 0.6.6). Quality of the sequencing reads (after trimming) were interrogated with FastQC (Andrews, 2014) (version 0.11.9) and found to be excellent. Reads were then aligned to the GENCODE release 31 GRCh38.p12 genome using the GENCODE v31 transcript annotation with the STAR (Dobin et al., 2013) aligner (version 2.7.6) using basic 2-pass mapping, with all 1st pass junctions inserted into the genome indices on the fly. Supplementary Table S1 indicates the number of reads successfully aligned for each of the samples.

Analysis and visualization of the resulting data were performed using R version 4.0.3 (R Foundation for Statistical Computing, Vienna, Austria). Aligned reads were quantified using the GenomicAlignments (Lawrence et al., 2013) package (Bioconductor) with the “IntersectionStrict” setting. Resulting read counts were log base two transformed and normalized using the rlog function of DESeq2 (Love et al., 2014). Additionally, DESeq2 was utilized to estimate linear regression models relating gene expression and NAT1 *N*-acetylation activity. This method allows us to identify genes whose expression is significantly associated with NAT1 *N*-acetylation activity. Significance of linear regression analysis is reported as adjusted (for multiple comparisons) Wald test p-values. Principal component analysis (PCA) was conducted by singular value decomposition of the centered data matrix on the most 1,000 variable genes in the entire dataset. The scores of the first (*x*-axis) and second (*y*-axis) principal component were plotted.

Log_2_ change in gene expression per unit change in NAT1 *N*-acetylation activity and significance (adjusted p-values) from regression analysis were visualized using volcano plots. Unbiased clustering of samples by hierarchal clustering was conducted using the WPGMA method using the most significant 100 differentially expressed genes; resulting data were plotted as heatmaps for visualization. Pathway enrichment analysis was conducted utilizing the linear regression results as input with the absolute value of the Wald test statistic as the variable for ordering. We utilized the normalized enrichment score to determine the relative degree of enrichment. Figure 1 illustrates the overall experimental approach and data analyses methods.

### 2.4 Quantitative Measurement of *NAT1* and *NAT2* mRNA

Quantitative Real-Time Reverse Transcription Polymerase Chain Reaction (qRT-PCR) was conducted for *NAT1* and arylamine N-acetyltransferase 2 (*NAT2*) in each of the six constructed cell lines as previously described (Husain et al., 2007a; Husain et al., 2007b; Millner et al., 2012). Briefly, total RNA was isolated from each cell line using the RNeasy Mini Kit (Qiagen, Germantown, MD). Isolated RNA was used to transcribe cDNA using the High Capacity Reverse Transcriptase kit (Life Technologies, Carlsbad, CA). Resulting cDNA was utilized for quantitative measurement of NAT1 and NAT2 via qRT-PCR. TaqMan analysis was performed using the ABI 7700 sequence detection system (Applied Biosystems, Foster City, CA). The gene probes utilized were designed previously to discriminate between *NAT1* and *NAT2*. Student’s t-tests were utilized for testing significance.

### 2.5 NAT2 *N*-Acetylation Activity Assays

*In vitro* NAT2 *N*-acetylation activity was determined in each constructed cell line as previously described (Grant et al., 1991; Hein et al., 1993). Briefly, cell lysate from each cell line was incubated with 1 mM acetyl-coenzyme A and 300 μM sulfamethazine (SMZ) at 37°C for 10 min. Reactions were terminated with the addition of 1/10 reaction volume 1 M acetic acid. Reaction products were collected and analyzed using an Agilent Technologies 1,260 Infinity high performance liquid chromatography (HPLC) using a LiChrospher 100 RP-18 (5 μm) column to determine the amount of acetylated product. Our reported limit of detection for *N*-acetylated SMZ is 0.15 nmol/min/mg.

## 3 Results

PABA *N*-acetylation activity was measured in constructed MDA-MB-231 cell lines and reported previously (Carlisle et al., 2020). Briefly, the *Scrambled* cell line had approximately the same activity as the *Parent* MDA-MB-231 cell line with no manipulation while the *Up* cell line had an approximate 700% increase in activity. Additionally, the *Down* and *CRISPR 2–12* cell lines had approximately 65 and 50% of the activity of the *Parent* and *Scrambled* cell lines, respectively, while the *CRISPR 2–19* and *CRISPR 5–50* cell lines had no detectable activity.

**FIGURE 2:**
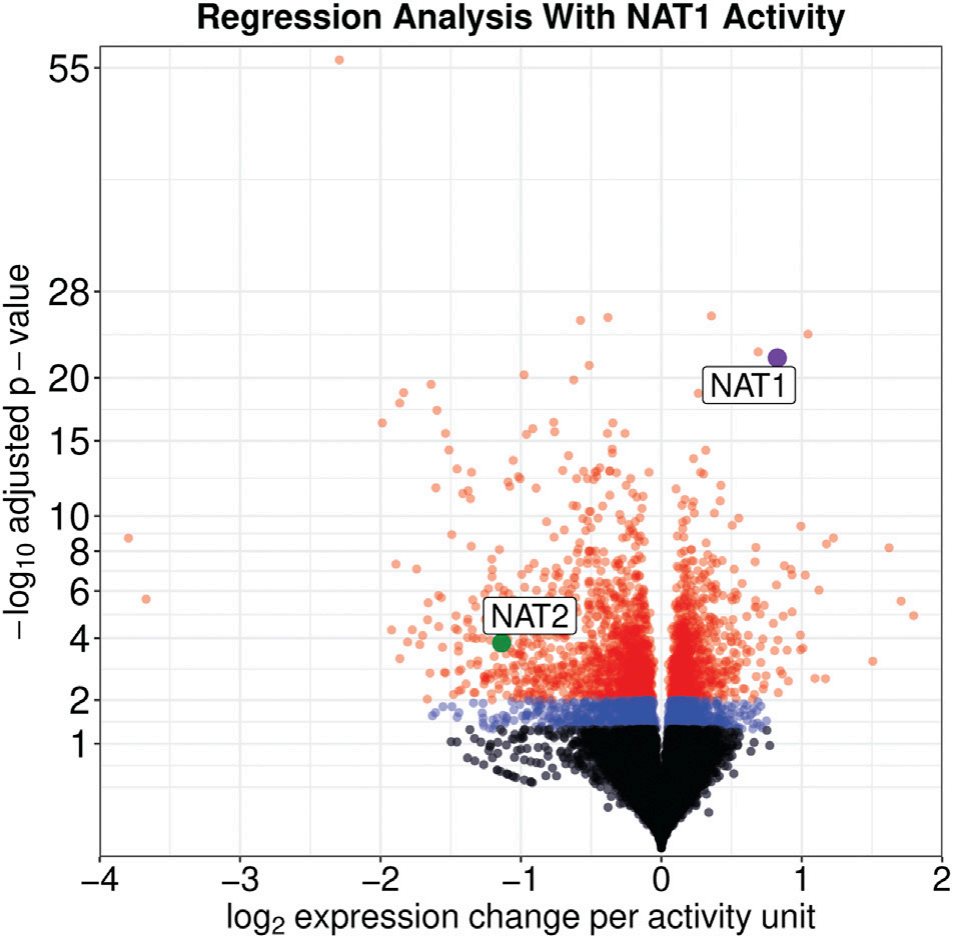
Expression of Many Genes, including NAT1 and NAT2, are significantly associated with NAT1 Activity. Significance and effect size are visualized for all genes in the dataset. Genes with an adjusted significance value greater than 0.05 are shown by black dots, genes with an adjusted significance value between 0.05 and 0.01 are shown by blue dots, and genes with an adjusted significance value less than 0.01 are shown by red dots. NAT1 is shown by a purple dot and NAT2 is shown by a green dot; both are labeled.

On a per-sample average, 91% of reads aligned to the human genome resulting in approximately 34.6 M uniquely aligned reads. Regression analysis revealed that the expression of 3,889 genes were significantly associated with the NAT1 *N*-acetylation activity of the cell lines (*adjusted p* ≤ 0.05); of those 3,889 genes, 1,756 were positively associated with NAT1 *N*-acetylation activity and 2,133 were negatively associated with NAT1 *N*-acetylation activity (Figure 2).

**FIGURE 3:**
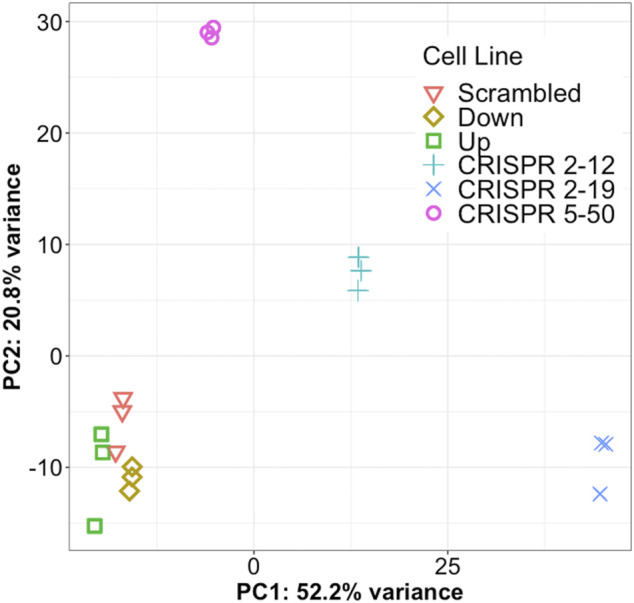
Each cell line has a unique global transcriptome. Principal component analysis, a statistical method for reducing the dimensionality of a dataset to better understand total variance, was conducted for all samples. Each symbol represents a single biological replicate. This analysis shows most of the total variance in the dataset (52.2%) is between the CRISPR-Cas9 generated cell lines from the other three cell lines. This analysis also shows each cell line has a distinct transcriptomic profile but the biological replicates are very similar to each other.

Principal component analysis of the 1,000 most variable genes in the dataset revealed that all cell lines in this study had distinct transcriptomic profiles as each clustered independently by group membership (Figure 3). In our dataset, principal component 1 explains 52.2% of the variance in the data while principal component 2 explains 20.8% of the variance. The *CRISPR 2–12, CRISPR 2–19,* and *CRISPR 5–50* groups are separated from the other three groups along principal component 1. The *CRISPR 2–12* and *CRISPR 5–50* cell lines were additionally separated from the remaining 4 cell lines along principal component 2. This revealed that the Scrambled, Up, and Down groups had transcriptomic profiles similar to each other but different from the other three groups suggesting the construction method utilized may have led to unique differences.

**FIGURE 4:**
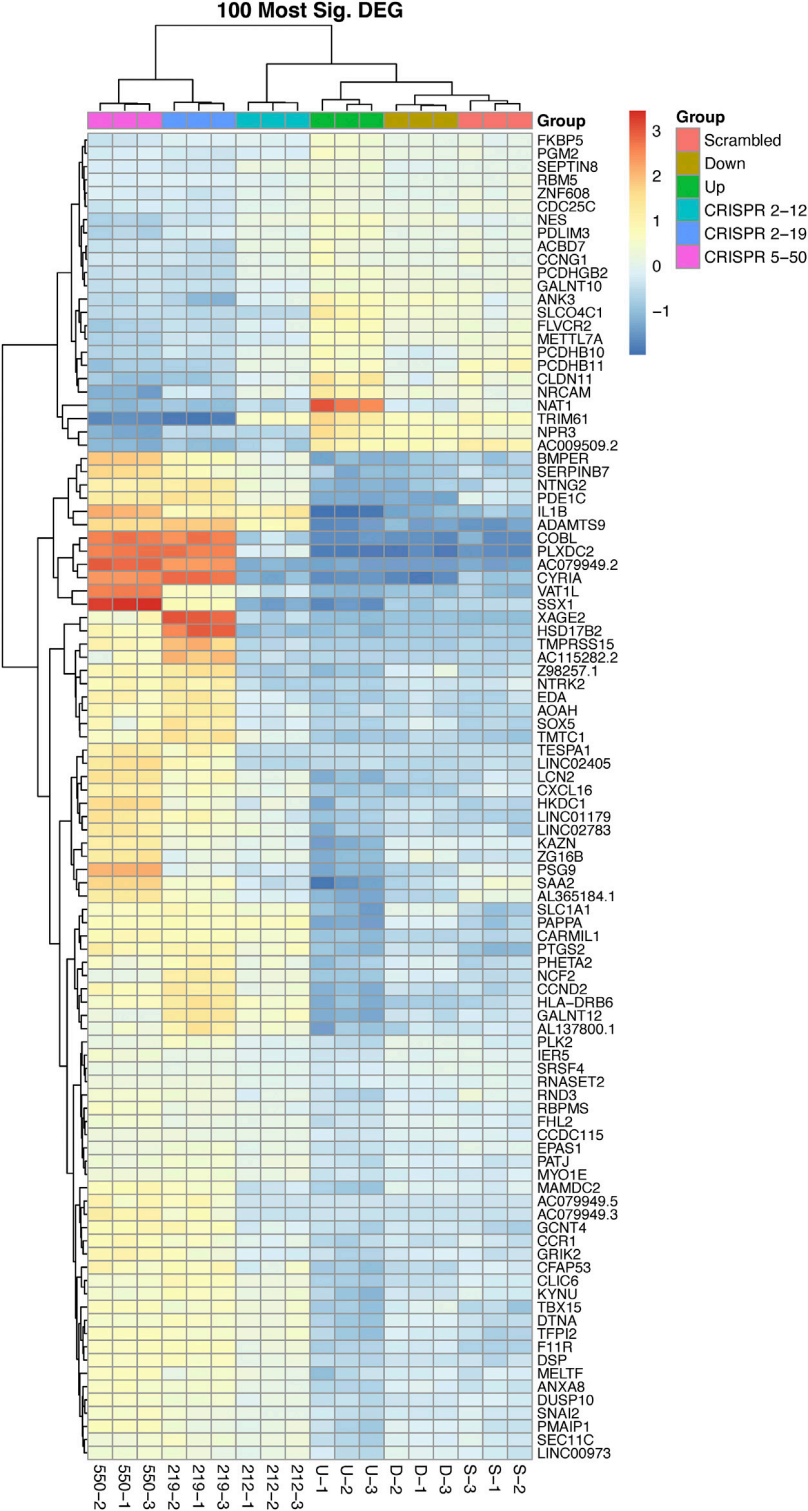
The 100 most differentially expressed genes cluster into two overall groups with distinguishable expression profiles. This analysis shows each cell line has a distinct expression profile for the 100 most significant genes associated with NAT1 activity and that there many genes with similar expression profiles across the samples. There are two overall groups that form in terms of similar patterns of expression, those that are positively associated with NAT1 activity (24—top cluster) and those that are negatively associated with NAT1 activity (76—bottom cluster). Each row of the heatmap shows the expression for a single gene while each column shows gene expression for an individual sample. Expression values have been transformed to Z-scores with red colors representing high expression and blue colors representing low expression.

Unsupervised hierarchical clustering of the 100 most significant genes associated with NAT1 *N*-acetylation activity was conducted to visualize gene expression patterns within all samples and to identify groups of genes with similar expression patterns (Figure 4). We observed one cluster of 24 genes, that included NAT1, to be positively associated with NAT1 *N*-acetylation activity. Gene expression in this cluster was generally low in the *CRISPR 2–19* and *CRISPR 5–50* cell lines, higher in the Down, *CRISPR 2–12*, and Scrambled cell line, and highest in the Up cell line. The remaining 76 genes which grouped as a separate cluster, exhibited the opposite pattern; gene expression in this cluster was generally highest in the *CRISPR 2–19* and *CRISPR 5–50* cell lines, lower in the Down, *CRISPR 2–12*, and Scrambled cell line, and lowest in the Up cell line.

Genes encoding proteins with functions related to acyl/acetyl transfer and binding were significantly associated with NAT1 *N*-acetylation activity. Vesicle amine transport 1 like (*VAT1L*) which is involved in acyl group transfer, was inversely related to NAT1 activity. Acyl-CoA binding domain containing 7 (*ACBD7*), was positively associated with NAT1 *N*-acetylation activity. Glucosaminyl (*N*-acetyl) transferase 4 (*GCNT4*) was negatively associated with NAT1 *N*-acetylation activity. Additionally, a negative association between 5-hydroxytryptamine receptor 1F (*HTR1F*) and NAT1 *N*-acetylation activity was observed.

Many protocadherin and cadherin genes were found to be significantly associated with NAT1 activity in the cell lines (Supplemental Table S2). Twenty-nine protocadherin genes were significantly associated with NAT1 activity (with no significant association observed in 33) and all but five of those genes were associated with an increase in expression per unit NAT1 activity. These included alpha, beta, and gamma subfamilies. Five cadherin genes (*CDH2*, *CDH3*, *CDH11*, *CDH13*, *CDH18*) were significantly associated with NAT1 activity and expression of all five were decreased per unit of NAT1 activity. Additionally, three of the four FAT atypical cadherin genes (*FAT1*, *FAT3*, *FAT4*) were significantly associated with NAT1 activity however the fold-changes were very small per NAT1 activity unit and in different directions; *FAT1* and *FAT4* were increased with NAT1 activity while *FAT3* was decreased with NAT1 activity. Transmembrane O-mannosyltransferase targeting cadherins 1 (*TMTC1*) was significantly associated with NAT1 activity and expression was decreased per unit NAT1 activity.

### 3.1 NAT Isozyme Expression in Transcriptomics Dataset

**FIGURE 5:**
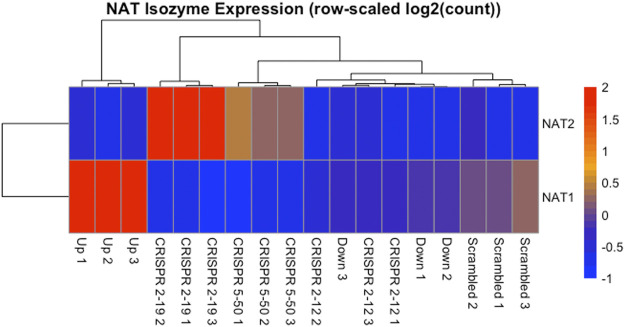
NAT Isozyme Expression is Inversely Related. The expression of NAT1 and its isozyme NAT2 were plotted as a heatmap for comparison. This data shows the inverse relationship between expression of NAT1 and NAT2 in each sample. When NAT1 transcript expression is high, NAT2 transcript expression is low, and conversely, when NAT1 transcript expression is low, NAT2 transcript expression is high. Each row of the heatmap shows the expression for a single gene while each column shows gene expression for an individual sample. Expression values have been transformed to Z-scores with red colors representing high expression and blue colors representing low expression.

**FIGURE 6:**
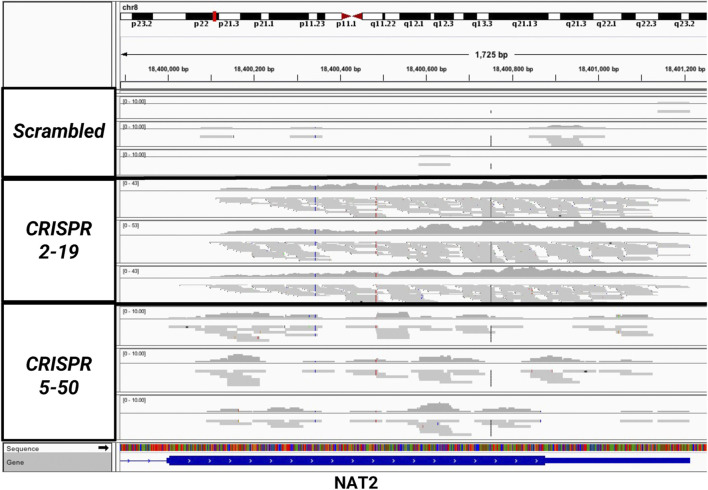
Sequencing Reads Map to the NAT2 Gene But Only in the Two Knockout Cell Lines, *CRISPR 2–19* and *CRISPR 5–50*. The mapping of reads to the NAT2 gene were visualized using the Integrated Genomic Viewer in the *Scrambled*, *CRISPR 2–19*, and *CRISPR 5–50* cell lines. Each linear gray bar represents an individual read. The red and blue lines represent positions in the read where a single nucleotide polymorphism compared to the reference genome is present. Zero to very few reads mapped to NAT2 in the *Scrambled* cell line samples, many reads mapped to NAT2 in the *CRISPR 2–19* cell line samples, and a medium amount of reads mapped to NAT2 in the *CRISPR 5–50* cell line samples.

**FIGURE 7:**
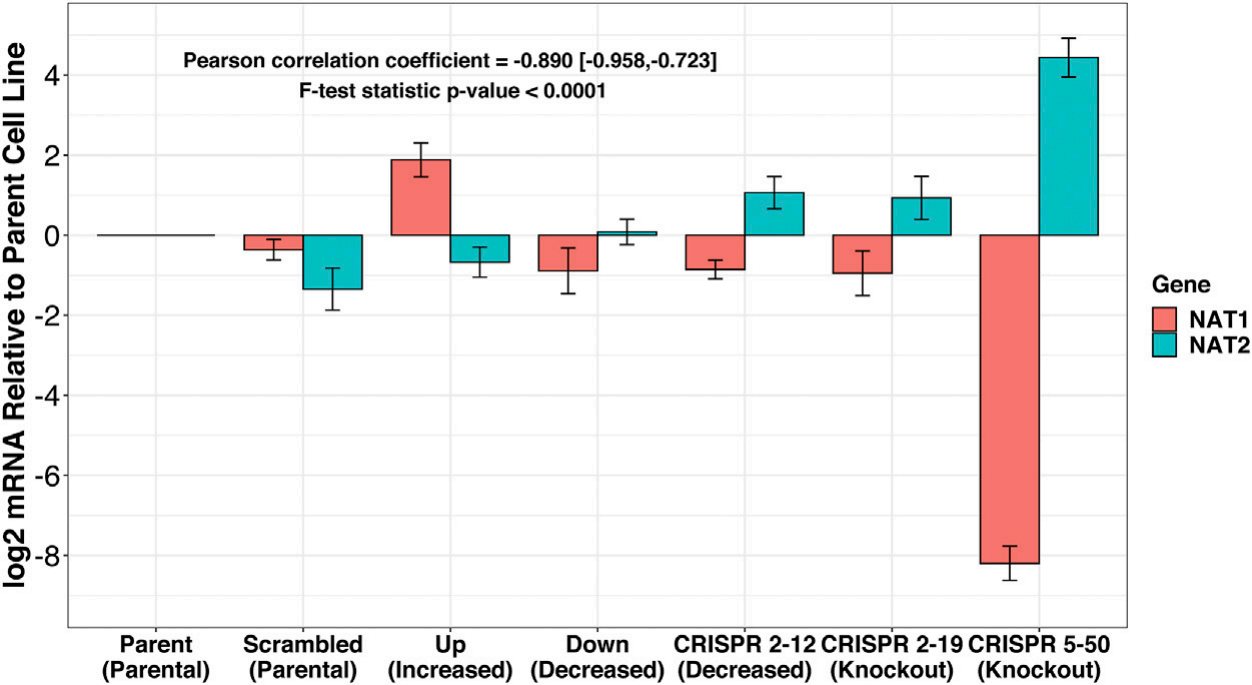
RT-qPCR confirms inverse relationship between NAT1 and NAT2 expression in our cell lines. PCR probes designed to discriminate between NAT1 and NAT2 were utilized for real-time quantitative polymerase chain reactions. mRNA expression was normalized to the expression of the parental MDA-MB-231 cell line. A trend of decreased NAT1 expression and increased NAT2 expression was observed. *p ≤ 0.05, ***p ≤ 0.001.

The expression of *NAT1* and its isozyme, *NAT2*, were compared between all cell lines. As expected, given the *N*-acetylation activities measured, NAT1 expression was decreased in the Down, *CRISPR 2–12*, *CRISPR 2–19*, and *CRISPR 5–50* cell lines and increased in the Up cell line compared to the Scrambled cell line. Notably, in the two complete NAT1 knockout cell lines (*CRISPR 2–19* and *CRISPR 5–50*), *NAT2* expression was observed while the expression was much lower in the other cell lines (Figure 5). We visualized the RNA-seq reads that were mapping to *NAT2* using the Integrated Genomics Viewer (IGV) to double check that the reads were not NAT1 reads mis-mapping to *NAT2* given the isozymes high degree of sequence homology (Figure 6); we confirmed these were not NAT1 misreads as we observed known NAT2 SNPs in the mapped sequence reads. The reads mapping to NAT2 had the following SNPs within them: rs1801280T > C and rs1799929C > T which corresponds with the NAT2*5A haplotype. We also verified the observation of NAT2 reads with qRT-PCR analysis for *NAT1* and *NAT2* in all 6 cell lines (Figure 7). Although not significant for all cell lines, we observed a pattern of decreased *NAT1* transcripts and increased *NAT2* transcripts in samples. We conducted NAT2 activity assays with cell lysate from all six constructed cell lines using the prototypic NAT2 substrate sulfamethazine (SMZ) however no NAT2 activity was detected in any cell line (data not shown). Currently, the functionality of the *NAT2* transcripts produced in the NAT1 knockout cell lines is unknown.

### 3.2 Pathway Analysis

**FIGURE 8:**
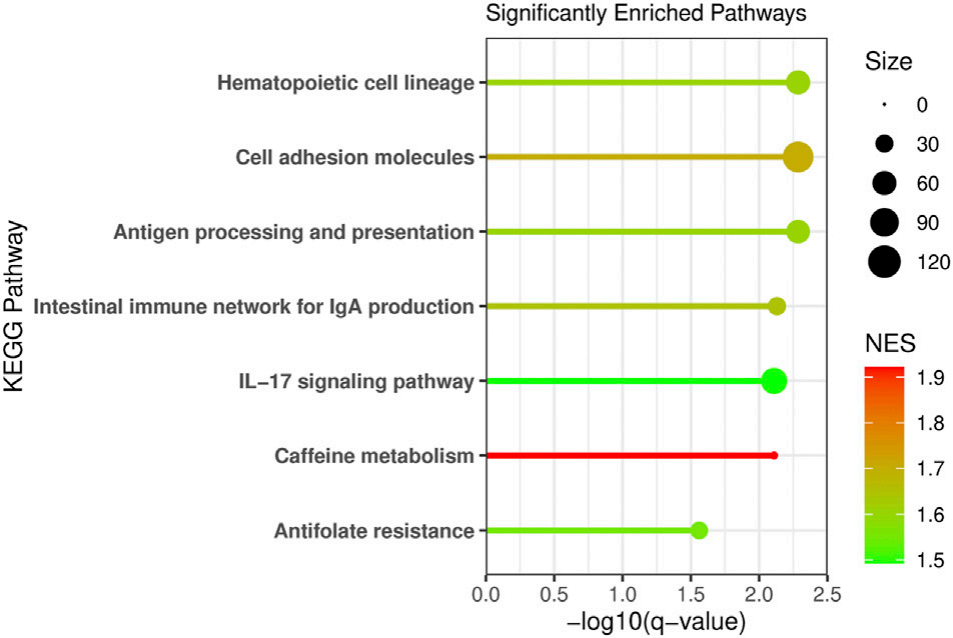
Pathway Analysis Reveals Enrichment of Specific Pathways for Differences Between Cell Lines. Pathway analysis was conducted using Gene Set Enrichment Analysis (GSEA) and the human Kyoto Encyclopedia of Genes and Genomes (KEGG) pathways. Genes were ranked by the absolute value of the Wald test statistic generated from the linear regression results. The KEGG pathway name is labeled on the *y*-axis, the −log10 of the q-value is plotted on the *x*-axis, the line and dot plotted are colored by the normalized enrichment score (NES) with the highest NES plotted in red and the lowest (from the significant pathways plotted) in green. The size of the dot in the figure represents the number of genes from our input that were in that specific pathway.

Pathway enrichment analysis was conducted using Gene Set Enrichment and the KEGG pathways to determine what metabolic pathways were enriched for genes associated with NAT1 activity (Figure 8). We focused on pathways that had a normalized enrichment score of >1.40. The Kyoto Encyclopedia of Genes and Genomes (KEGG) pathways were used (Ogata et al., 1999; Kanehisa and Goto, 2000). Disease associated pathways were removed from the analysis results. Many pathways were significantly enriched for differences including hematopoietic cell lineage, cell adhesion molecules, antigen processing and presentation, IL-17 signaling, intestinal immune network for IGA production, caffeine metabolism, and antifolate resistance pathways were found to be significantly enriched. This further supports our observation that NAT1 activity is strongly associated with the expression of cell adhesion genes. Additionally, pathways that we would expect to be affected by NAT1 given previous literature and knowledge, such as caffeine metabolism and antifolate resistance, were found to be significantly enriched for differences. Some of the enriched pathways additionally suggest a relationship between the immune system and NAT1.

## 4 Discussion

As our samples for this study were genetically modified breast cancer cell lines that had been passaged for a few growth cycles, we decided to utilize a regression approach for data analysis rather than pair-wise comparisons to our control cell line. This approach decreases the likelihood that genes identified to be differentially expressed between groups are due to off-target effects of CRISPR/Cas9 editing and/or additional mutations incurred during passaging of cell lines or during clonal selection after CRISPR/Cas9. One limitation of this approach, however, is that it relies on NAT1 *N*-acetyltransferase activity being the functional measure for this enzyme’s activity and may limit us identifying genes that are affected if NAT1 has any enzyme functions independent of this activity. Nevertheless, we should still be able to detect some of those possible independent effects because we know our complete NAT1 knockout cell lines have mutations that lead to very truncated NAT1 protein that have lost nearly all protein domains. Additionally, it has been noted by others that CRISPR/Cas9 genetic editing frequently leads to on-target mRNA misregulation which leads to degradation of the mutant mRNAs produced (Tuladhar et al., 2019).

In recent years many roles for NAT1 independent of *N*-acetylation of exogenous xenobiotics have been shown. One challenge of identifying all of the potential roles of NAT1 in metabolism has been the approaches taken thus far. When performing targeted assays probing NAT1’s effect on specific pathways/genes we are limited by what is known when deciding where to look. In this study we have taken a global systems biology approach which removes that previous limitation.

In this study we identified additional supportive evidence of an association between NAT1 and cell adhesion molecules. Many have noted that overexpression NAT1, specifically in breast cancer, is associated with increased metastasis (Smid et al., 2006; Savci-Heijink et al., 2016; Zhao et al., 2020) and growth (Adam et al., 2003) and conversely knockdown or deletion of NAT1 leads to increased adherence to collagen (Li et al., 2020) and decreased invasion (Tiang et al., 2010, 2015; Li et al., 2020), E-cadherin up-regulation and cell-cell contact growth inhibition (Tiang et al., 2011), and reduced anchorage independent growth (Stepp et al., 2018; Stepp et al., 2019). Here we have shown the expression of many members of the cadherin family of proteins are associated with NAT1 *N*-acetylation activity. Particularly, the negative association between the genes *CDH2*, *CDH3*, *CDH11*, *CDH13*, *CDH18* and NAT1 *N*-acetylation activity supports observations of high NAT1 expression in breast cancer and increased metastasis.

The cadherin family of genes are subject to numerous regulatory mechanisms (Gumbiner, 2000) complicating the interpretation of the relationship between cadherins and NAT1 activity directly. However, we additionally observed that expression of *TMTC1*, a gene that encodes a protein important for adding O-mannosyl post-translational modifications to members of the cadherin family, was inversely related to NAT1 activity; it has been shown by others that TMTC3, a closely related family member to TMTC1, is important for ensuring E-cadherin operates properly in cell adhesion (Graham et al., 2020); when TMTC1 was knocked out cells showed increased migration ability (Graham et al., 2020). Our pathway analysis indicated the KEGG pathway: cell adhesion molecules, was one of the most significantly enriched pathways for genes associated with NAT1 activity. Although the mechanism behind this relationship remains unclear, this adds additional evidence of a relationship and further impetus to study this phenomenon more closely in order to potentially elucidate NAT1 as an anti-metastasis drug target.

The observation that the complete knockout, but not knockdown, of NAT1 lead to NAT2 transcript production leads us to hypothesize that there may be a compensation mechanism that occurs when NAT1 *N*-acetylation is lost, suggesting NAT1 has an essential role. There have been examples of humans that have no detectable NAT1 *N*-acetylation activity (Bruhn et al., 1999) however NAT2 expression and/or activity has never been investigated specifically in this population. Notably, a retrospective analysis of publicly available NAT1 and NAT2 gene expression data in established breast cancer cell lines, primary breast tumor tissues, and normal breast tissues showed a small positive correlation between the two genes (Carlisle and Hein, 2018). One reason that study did not observe an inverse relationship between NAT1 and NAT2 gene expression may be because complete knockout of NAT1 may be necessary before the compensation mechanism occurs given we only observed NAT2 transcripts in the complete NAT1 KO cell lines but not the cell lines with decreased NAT1. Data reported by the Cancer Cell Line Encyclopedia (CCLE) indicates the MDA-MB-231 cell line is diploid for NAT2 and does not show copy number variation (Ghandi et al., 2019).

The relationship between NAT1 and NAT2 expression has not been studied in-depth although it is known each has tissue specific expression. NAT1 is expressed throughout most tissues while NAT2 is most highly expressed in the liver and digestive tract. Since NAT1 and NAT2 are isozymes with overlapping substrate specificities (although distinct) it has been thought that the isozyme with the highest expression would be most important to understand in a given tissue. While our RNA-seq data showed the highest levels of NAT2 transcripts in the *CRISPR 2–19* cell line, our qRT-PCR showed the highest levels of NAT2 transcripts in the *CRISPR 5–50* cell line. We hypothesize that this is because the samples for each set of experiments were collected at different times; the phase of cell growth as well as growth conditions may affect the mechanism responsible for transcription of *NAT2*. Additionally, others have noted that CRISPR/Cas9 genetic editing can lead to degradation of the mutant mRNAs produced (*NAT1* in our experiments); we hypothesize that this process may also be affected by the growth conditions or growth phase during sample collection.

Additionally, the associations observed between NAT1 *N*-acetylation activity and other genes closely related to transfer of acyl groups such as, *VAT1L* (inversely related to NAT1 activity), *GCNT4* (negatively associated with NAT1 *N*-acetylation activity), and *ACBD7* (positively associated with NAT1 *N*-acetylation activity) suggest there are mechanisms occurring to maintain homeostasis as it relates to acetyl group transfers. *ACBD7* is a member of a family of proteins involved in numerous intracellular processes including fatty acid-, glycerolipid- and glycerophospholipid biosynthesis, *β*-oxidation, cellular differentiation and proliferation as well as in the regulation of numerous enzyme activities (Neess et al., 2015). This association may help us better understand the finding in our previous metabolomics study that the *β*-oxidation of fatty acids seemed to be impaired in NAT1 deficient cell lines (Carlisle et al., 2020).

As NAT1 is known to *N*-acetylate serotonin (Steinberg et al., 1969; Backlund et al., 2017), albeit at very low levels in comparison to aralkylamine N-acetyltransferase (AANAT), we find the negative association between 5-hydroxytryptamine receptor 1F (*HTR1F*) and NAT1 *N*-acetylation activity interesting. The negative association between *HTR1F* and NAT1 *N*-acetylation activity indicates an upregulation of the serotonin receptor when NAT activity is low and presumably less serotonin is N-acetylated and a downregulation of the serotonin receptor when NAT activity is high and presumably more serotonin is N-acetylated. In our previous metabolomics study of the same samples, a positive association was observed between NAT1 *N*-acetylation activity and serotonin metabolite abundance (Carlisle et al., 2020). Taken together, this study suggests there are complex homeostatic/compensatory mechanisms occurring based on NAT1 levels as well as other factors such as AANAT levels.

## 5 Conclusion

This is the first study to measure the global transcriptome profile of breast cancer cells with varying levels of NAT1 *N*-acetylation activity. The expression of many genes, especially genes in the cadherin family, were significantly associated with NAT1 *N*-acetylation activity suggesting either regulation by NAT1, regulation by acetyl-CoA (or products of its hydrolysis), co-regulation mechanisms, or mechanisms compensating for the loss of NAT1. The most notable finding of this study was the observation that *NAT2* gene expression was significantly inversely associated with NAT1 *N*-acetylation activity and this relationship was also observed via RT-PCR methods. It appears only the complete knockout of NAT1 stimulates transcription of *NAT2* suggesting complete loss of NAT1 *N*-acetylation activity is necessary. This observation suggests *NAT2* transcripts are produced as a compensation mechanism for the complete loss of *NAT1* however the mechanism by which the cell senses the loss of *NAT1* and transcribes *NAT2* has not been identified and requires further investigation. As the two NAT1 knockout cell lines (nor any of the other cell lines) did not have any detectable *N*-acetylation activity toward the prototypic NAT2 substrate SMZ (limit of detection = 0.15 nmol/min/mg), the functionality of the *NAT2* transcripts remains unknown and warrants further investigation.

## Data Availability

The original contributions presented in the study are publicly available. This data can be found here: https://www.ncbi.nlm.nih.gov/Traces/study/?acc=PRJNA774803.

## References

[B1] Adam P. J. Berry J. Loader J. A. Tyson K. L. Craggs G. Smith P. (2003). Arylamine N-Acetyltransferase-1 Is Highly Expressed in Breast Cancers and Conveys Enhanced Growth and Resistance to Etoposide *In Vitro* . Mol. Cancer Res. 1 (11), 826–835. 14517345

[B2] Andrews S. (2014). FastQC: A Quality Control Tool for High Throughput Sequence Data [Online]. Available: http://bioinformatics.babraham.ac.uk/projects/fastqc/ (Accessed October 14, 2021).

[B3] Backlund P. S. Urbanski H. F. Doll M. A. Hein D. W. Bozinoski M. Mason C. E. (2017). Daily Rhythm in Plasma N-Acetyltryptamine. J. Biol. Rhythms 32 (3), 195–211. 10.1177/0748730417700458 28466676 PMC5571864

[B4] Bruhn C. Brockmöller J. Cascorbi I. Roots I. Borchert H. H. (1999). Correlation between Genotype and Phenotype of the Human Arylamine N-Acetyltransferase Type 1 (NAT1). Biochem. Pharmacol. 58 (11), 1759–1764. 10.1016/s0006-2952(99)00269-5 10571250

[B5] Carlisle S. Hein D. (2018). Retrospective Analysis of Estrogen Receptor 1 and N‑acetyltransferase Gene Expression in normal Breast Tissue, Primary Breast Tumors, and Established Breast Cancer Cell Lines. Int. J. Oncol. 53, 694-702. 10.3892/ijo.2018.4436 29901116 PMC6017241

[B6] Carlisle S. M. Trainor P. J. Doll M. A. Stepp M. W. Klinge C. M. Hein D. W. (2018). Knockout of Human Arylamine N-Acetyltransferase 1 (NAT1) in MDA-MB-231 Breast Cancer Cells Leads to Increased reserve Capacity, Maximum Mitochondrial Capacity, and Glycolytic reserve Capacity. Mol. Carcinog 57 (11), 1458–1466. 10.1002/mc.22869 29964355 PMC6353662

[B7] Carlisle S. M. Trainor P. J. Hong K. U. Doll M. A. Hein D. W. (2020). CRISPR/Cas9 Knockout of Human Arylamine N-Acetyltransferase 1 in MDA-MB-231 Breast Cancer Cells Suggests a Role in Cellular Metabolism. Sci. Rep. 10 (1), 9804. 10.1038/s41598-020-66863-4 32555504 PMC7299936

[B8] Cock P. J. Fields C. J. Goto N. Heuer M. L. Rice P. M. (2010). The Sanger FASTQ File Format for Sequences with Quality Scores, and the Solexa/Illumina FASTQ Variants. Nucleic Acids Res. 38 (6), 1767–1771. 10.1093/nar/gkp1137 20015970 PMC2847217

[B9] Dobin A. Davis C. A. Schlesinger F. Drenkow J. Zaleski C. Jha S. (2013). STAR: Ultrafast Universal RNA-Seq Aligner. Bioinformatics 29 (1), 15–21. 10.1093/bioinformatics/bts635 23104886 PMC3530905

[B10] Ghandi M. Huang F. W. Jané-Valbuena J. Kryukov G. V. Lo C. C. McDonald E. R. 3rd (2019). Next-generation Characterization of the Cancer Cell Line Encyclopedia. Nature 569 (7757), 503–508. 10.1038/s41586-019-1186-3 31068700 PMC6697103

[B11] Graham J. B. Sunryd J. C. Mathavan K. Weir E. Larsen I. S. B. Halim A. (2020). Endoplasmic Reticulum Transmembrane Protein TMTC3 Contributes to O-Mannosylation of E-Cadherin, Cellular Adherence, and Embryonic Gastrulation. Mol. Biol. Cel 31 (3), 167–183. 10.1091/mbc.E19-07-0408 PMC7001481 31851597

[B12] Grant D. M. Blum M. Beer M. Meyer U. A. (1991). Monomorphic and Polymorphic Human Arylamine N-Acetyltransferases: a Comparison of Liver Isozymes and Expressed Products of Two Cloned Genes. Mol. Pharmacol. 39 (2), 184–191. 1996083

[B13] Gumbiner B. M. (2000). Regulation of Cadherin Adhesive Activity. J. Cel Biol 148 (3), 399–404. 10.1083/jcb.148.3.399 PMC2174801 10662767

[B14] Hein D. W. Doll M. A. Rustan T. D. Gray K. Feng Y. Ferguson R. J. (1993). Metabolic Activation and Deactivation of Arylamine Carcinogens by Recombinant Human NAT1 and Polymorphic NAT2 Acetyltransferases. Carcinogenesis 14 (8), 1633–1638. 10.1093/carcin/14.8.1633 8353847

[B15] Husain A. Zhang X. Doll M. A. States J. C. Barker D. F. Hein D. W. (2007a). Functional Analysis of the Human N-Acetyltransferase 1 Major Promoter: Quantitation of Tissue Expression and Identification of Critical Sequence Elements. Drug Metab. Dispos 35 (9), 1649–1656. 10.1124/dmd.107.016485 17591675 PMC2085369

[B16] Husain A. Zhang X. Doll M. A. States J. C. Barker D. F. Hein D. W. (2007b). Identification of N-Acetyltransferase 2 (NAT2) Transcription Start Sites and Quantitation of NAT2-specific mRNA in Human Tissues. Drug Metab. Dispos 35 (5), 721–727. 10.1124/dmd.106.014621 17287389 PMC1931608

[B17] Kanehisa M. Goto S. (2000). KEGG: Kyoto Encyclopedia of Genes and Genomes. Nucleic Acids Res. 28 (1), 27–30. 10.1093/nar/28.1.27 10592173 PMC102409

[B18] Laurieri N. Dairou J. Egleton J. E. Stanley L. A. Russell A. J. Dupret J. M. (2014). From Arylamine N-Acetyltransferase to Folate-dependent Acetyl CoA Hydrolase: Impact of Folic Acid on the Activity of (HUMAN)NAT1 and its Homologue (MOUSE)NAT2. PLoS One 9 (5), e96370. 10.1371/journal.pone.0096370 24823794 PMC4019507

[B19] Lawrence M. Huber W. Pagès H. Aboyoun P. Carlson M. Gentleman R. (2013). Software for Computing and Annotating Genomic Ranges. Plos Comput. Biol. 9 (8), e1003118. 10.1371/journal.pcbi.1003118 23950696 PMC3738458

[B20] Li P. Butcher N. J. Minchin R. F. (2020). Effect Arylamine N-Acetyltransferase 1 on Morphology, Adhesion, Migration, and Invasion of MDA-MB-231 Cells: Role of Matrix Metalloproteinases and Integrin αV. Cell Adh Migr 14 (1), 1–11. 10.1080/19336918.2019.1710015 31910058 PMC6961680

[B21] Love M. I. Huber W. Anders S. (2014). Moderated Estimation of Fold Change and Dispersion for RNA-Seq Data with DESeq2. Genome Biol. 15 (12), 550. 10.1186/s13059-014-0550-8 25516281 PMC4302049

[B22] Millner L. M. Doll M. A. Stepp M. W. States J. C. Hein D. W. (2012). Functional Analysis of Arylamine N-Acetyltransferase 1 (NAT1) NAT1*10 Haplotypes in a Complete NATb mRNA Construct. Carcinogenesis 33 (2), 348–355. 10.1093/carcin/bgr273 22114069 PMC3271262

[B23] Minchin R. F. Butcher N. J. (2018). Trimodal Distribution of Arylamine N-Acetyltransferase 1 mRNA in Breast Cancer Tumors: Association with Overall Survival and Drug Resistance. BMC Genomics 19 (1), 513. 10.1186/s12864-018-4894-4 29969986 PMC6029418

[B24] Neess D. Bek S. Engelsby H. Gallego S. F. Færgeman N. J. (2015). Long-chain Acyl-CoA Esters in Metabolism and Signaling: Role of Acyl-CoA Binding Proteins. Prog. Lipid Res. 59, 1–25. 10.1016/j.plipres.2015.04.001 25898985

[B25] Ogata H. Goto S. Sato K. Fujibuchi W. Bono H. Kanehisa M. (1999). KEGG: Kyoto Encyclopedia of Genes and Genomes. Nucleic Acids Res. 27 (1), 29–34. 10.1093/nar/27.1.29 9847135 PMC148090

[B26] Savci-Heijink C. D. Halfwerk H. Koster J. van de Vijver M. J. (2016). A Novel Gene Expression Signature for Bone Metastasis in Breast Carcinomas. Breast Cancer Res. Treat. 156 (2), 249–259. 10.1007/s10549-016-3741-z 26965286 PMC4819548

[B27] Siegel R. L. Miller K. D. Jemal A. (2018). Cancer Statistics, 2018. CA Cancer J. Clin. 68 (1), 7–30. 10.3322/caac.21442 29313949

[B28] Siegel R. L. Miller K. D. Fuchs H. E. Jemal A. (2021). Cancer Statistics, 2021. CA A. Cancer J. Clin. 71 (1), 7–33. 10.3322/caac.21654 33433946

[B29] Smid M. Wang Y. Klijn J. G. Sieuwerts A. M. Zhang Y. Atkins D. (2006). Genes Associated with Breast Cancer Metastatic to Bone. J. Clin. Oncol. 24 (15), 2261–2267. 10.1200/JCO.2005.03.8802 16636340

[B30] Steinberg M. S. Cohen S. N. Weber W. W. (1969). Acetylation of 5-hydroxytryptamine by Isoniazid N-Acetyltransferase. Biochim. Biophys. Acta 184 (1), 210–212. 10.1016/0304-4165(69)90119-6 5791110

[B31] Stepp M. W. Doll M. A. Carlisle S. M. States J. C. Hein D. W. (2018). Genetic and Small Molecule Inhibition of Arylamine N-Acetyltransferase 1 Reduces anchorage-independent Growth in Human Breast Cancer Cell Line MDA-MB-231. Mol. Carcinog 57 (4), 549–558. 10.1002/mc.22779 29315819 PMC5832614

[B32] Stepp M. W. Doll M. A. Samuelson D. J. Sanders M. A. States J. C. Hein D. W. (2017). Congenic Rats with Higher Arylamine N-Acetyltransferase 2 Activity Exhibit Greater Carcinogen-Induced Mammary Tumor Susceptibility Independent of Carcinogen Metabolism. BMC Cancer 17 (1), 233. 10.1186/s12885-017-3221-9 28359264 PMC5374573

[B33] Stepp M. W. Mamaliga G. Doll M. A. States J. C. Hein D. W. (2015). Folate-Dependent Hydrolysis of Acetyl-Coenzyme A by Recombinant Human and Rodent Arylamine N-Acetyltransferases. Biochem. Biophys. Rep. 3, 45–50. 10.1016/j.bbrep.2015.07.011 26309907 PMC4545580

[B34] Stepp M. W. Salazar-Gonzalez R. A. Hong K. U. Doll M. A. Hein D. W. (2019). N-Acetyltransferase 1 Knockout Elevates Acetyl Coenzyme A Levels and Reduces Anchorage-Independent Growth in Human Breast Cancer Cell Lines. J. Oncol. 2019, 3860426. 10.1155/2019/3860426 31531019 PMC6720663

[B35] Tiang J. M. Butcher N. J. Cullinane C. Humbert P. O. Minchin R. F. (2011). RNAi-Mediated Knock-Down of Arylamine N-Acetyltransferase-1 Expression Induces E-Cadherin Up-Regulation and Cell-Cell Contact Growth Inhibition. PLoS One 6 (2), e17031. 10.1371/journal.pone.0017031 21347396 PMC3036737

[B36] Tiang J. M. Butcher N. J. Minchin R. F. (2015). Effects of Human Arylamine N-Acetyltransferase I Knockdown in Triple-Negative Breast Cancer Cell Lines. Cancer Med. 4 (4), 565–574. 10.1002/cam4.415 25627111 PMC4402071

[B37] Tiang J. M. Butcher N. J. Minchin R. F. (2010). Small Molecule Inhibition of Arylamine N-Acetyltransferase Type I Inhibits Proliferation and Invasiveness of MDA-MB-231 Breast Cancer Cells. Biochem. Biophys. Res. Commun. 393 (1), 95–100. 10.1016/j.bbrc.2010.01.087 20100460

[B38] Tuladhar R. Yeu Y. Tyler Piazza J. Tan Z. Rene Clemenceau J. Wu X. (2019). CRISPR-Cas9-based Mutagenesis Frequently Provokes On-Target mRNA Misregulation. Nat. Commun. 10 (1), 4056. 10.1038/s41467-019-12028-5 31492834 PMC6731291

[B39] Zhang X. Carlisle S. M. Doll M. A. Martin R. C. G. States J. C. Klinge C. M. (2018). High N-Acetyltransferase 1 Expression Is Associated with Estrogen Receptor Expression in Breast Tumors, but Is Not under Direct Regulation by Estradiol, 5α-Androstane-3β, 17β-Diol, or Dihydrotestosterone in Breast Cancer Cells. J. Pharmacol. Exp. Ther. 365, 84–93. 10.1124/jpet.117.247031 29339455 PMC5830641

[B40] Zhao C. Cai X. Wang Y. Wang D. Wang T. Gong H. (2020). NAT1 Promotes Osteolytic Metastasis in Luminal Breast Cancer by Regulating the Bone Metastatic Niche via NF-κB/IL-1B Signaling Pathway. Am. J. Cancer Res. 10 (8), 2464–2479. 32905535 PMC7471372

