# Supplementary material for: Human Arylamine N-Acetyltransferase 1 (NAT1) Knockout in MDA-MB-231 Breast Cancer Cell Lines Leads to Transcription of NAT2
Source: Front Pharmacol. 2022 Jan 3;12:803254. doi: 10.3389/fphar.2021.803254 (PMC8762260; doi:10.3389/fphar.2021.803254)
Supplement: Supplementary file 2 [file Table1.DOCX]

**Supplementary Table 1: Mapping Statistics**

Mapping rates, millions of reads aligned, percent of duplicate reads, percent GC content, and millions of sequences generated were calculated for each sample.

| **Sample Name** | **% Aligned** | **M Aligned** | **% Dups** | **% GC** | **M Seqs** |
| --- | --- | --- | --- | --- | --- |
| Scrambled 1 | 89.30% | 33.9 | 62.90% | 45% | 38.0 |
| Scrambled 2 | 90.40% | 31.9 | 61.80% | 44% | 35.4 |
| Scrambled 3 | 89.50% | 34.2 | 62.80% | 42% | 38.2 |
| Down 1 | 91.00% | 37.6 | 64.60% | 42% | 41.3 |
| Down 2 | 91.00% | 37.6 | 65.40% | 41% | 41.3 |
| Down 3 | 90.60% | 40.6 | 64.50% | 42% | 44.8 |
| Up 1 | 91.50% | 34.9 | 64.90% | 40% | 38.2 |
| Up 2 | 90.30% | 33.7 | 60.80% | 44% | 37.3 |
| Up 3 | 90.50% | 35.3 | 60.80% | 45% | 39.0 |
| CRISPR 2-12 1 | 90.70% | 30.2 | 58.40% | 45% | 33.3 |
| CRISPR 2-12 2 | 91.00% | 35.1 | 60.30% | 44% | 38.6 |
| CRISPR 2-12 3 | 90.70% | 34.7 | 61.40% | 43% | 38.3 |
| CRISPR 2-19 1 | 90.90% | 35.3 | 62.80% | 44% | 38.9 |
| CRISPR 2-19 2 | 91.20% | 31.7 | 62.00% | 42% | 34.8 |
| CRISPR 2-12 3 | 91.40% | 31.2 | 60.80% | 44% | 34.1 |
| CRISPR 5-50 1 | 89.90% | 31.8 | 60.80% | 45% | 35.4 |
| CRISPR 5-50 2 | 90.10% | 38.3 | 62.10% | 44% | 42.5 |
| CRISPR 5-50 3 | 90.30% | 34.1 | 62.90% | 44% | 37.7 |
